# Performance of Fly Ash-Blended Cementitious Systems Incorporating CaCl_2_ and TEA: Effects on Mechanical and Hydration Properties

**DOI:** 10.3390/ma19214480

**Published:** 2026-10-24

**Authors:** Yusuf Çetinkaya, Muhammet Gökhan Altun

**Affiliations:** Department of Civil Engineering, Çanakkale Onsekiz Mart University, Çanakkale 17000, Türkiye; yusufcetinkaya650@gmail.com

**Keywords:** cement-based systems, fly ash, set-accelerating admixture, triethanolamine

## Abstract

This study experimentally investigates the effects of CaCl_2_ and TEA, used individually and in combination, on the fresh and hardened properties of fly ash-blended cementitious systems. The primary objective was to evaluate the potential of chemical admixtures to mitigate the setting retardation and insufficient early-age strength development associated with fly ash incorporation while examining the effects of different admixture dosages. Cementitious mixtures containing 20% fly ash, CaCl_2_ at 1%, 3%, and 5%, and TEA at 0.1%, 0.2%, and 0.3% were prepared. Setting time, flow spread, compressive strength, and water absorption were determined, and the experimental data were evaluated using ANOVA. The results showed that fly ash incorporation prolonged setting time and limited early-age strength development. CaCl_2_ significantly reduced the setting time with increasing dosage, mitigating the retarding effect of fly ash; however, higher dosages markedly reduced fresh-state flowability. TEA used alone prolonged setting time, particularly at higher dosages, whereas its retarding effect was substantially moderated when combined with CaCl_2_. Nevertheless, the combined use of CaCl_2_ and TEA did not provide the expected improvement in mechanical performance, and particularly at higher dosages, resulted in significant reductions in compressive strength.

## 1. Introduction

The construction sector is a rapidly evolving industry that addresses the requirements of industrialization and infrastructure development and plays a central role in economic growth. Concrete is undoubtedly an integral component of this sector and is regarded as the most widely consumed basic construction material worldwide [1,2]. Although Ordinary Portland Cement (OPC) is the primary binding component in concrete, the clinker production process is one of the major sources of greenhouse gas emissions directly associated with global warming and climate change [3]. Accordingly, the development of low-carbon and sustainable construction materials has become imperative to mitigate the environmental impacts associated with the cement industry and to reduce the enormous energy consumption required for clinker calcination [1,2]. In this context, the partial replacement of cement with mineral additives, commonly referred to as Supplementary Cementitious Materials (SCMs), is considered one of the most promising approaches to meeting sustainability requirements and reducing the carbon footprint of concrete production [3,4]. These mineral additives, which are primarily derived from industrial by-products such as fly ash, ground granulated blast-furnace slag, and silica fume, have the potential to enhance the durability, workability, sulfate resistance, and service life of concrete by filling voids within the cementitious matrix and promoting the formation of additional hydration products [1,2].

Fly ash is an industrial by-product generated from the combustion of coal in thermal power plants and is among the most widely used replacement materials in the cement industry worldwide. Its incorporation into concrete production is primarily attributed to its strong pozzolanic activity, through which it reacts with the calcium hydroxide (CH) released during cement hydration, resulting in the formation of additional calcium silicate hydrate (C–S–H) gels [1,5,6,7]. This pozzolanic reaction fills the capillary pores within the concrete matrix, leading to a denser and more compact microstructure. Consequently, it contributes to the enhancement of long-term strength while simultaneously providing an environmentally beneficial solution to the problem of industrial waste management [1,8]. Despite these advantages, the use of fly ash at high replacement levels in cementitious systems introduces several significant limitations concerning hydration processes. The chemical composition of the glassy surface layer of fly ash acts as a barrier, resulting in a considerably slow pozzolanic reaction at early ages. This sluggish hydration kinetics leads to substantially lower early-age compressive strength in fly ash-containing cementitious systems compared with conventional cement systems [5,9,10]. Overcoming the insufficient early-age strength development and workability-related challenges associated with fly ash utilization is therefore critical for improving production efficiency in construction applications. These limitations are commonly addressed through the incorporation of chemical admixtures [8,11].

Chemical admixtures are materials added to concrete or mortar mixtures, in very small quantities apart from water, cement, and aggregates, to impart specific physical and mechanical properties that cannot be achieved with conventional concrete mixtures [12]. The role of these admixtures in concrete technology is highly diverse. They perform functions such as controlling setting time, reducing water demand, improving workability, modifying viscosity, and enhancing strength properties [11,13]. In cementitious systems, chemical admixtures directly and significantly alter the reaction kinetics by affecting the surface tension of cement particles and the rate of precipitation of hydration products. Setting accelerators are widely used in the concrete industry for various purposes. In shotcrete applications, they are employed to ensure safe application of the material, facilitate rapid setting, and promote early strength development. In conventional concrete, they are used for emergency repair works, rapid production to meet increasing demand, and concrete placement under low-temperature conditions. In 3D-printed concrete, setting accelerators are utilized to provide the necessary stabilization of the printed material. Furthermore, when low-clinker-content cements are used to reduce CO_2_ emissions, these admixtures are employed to sufficiently enhance the reaction rate and strength development [14]. Particularly in cold-weather concreting or emergency repair applications requiring high early strength, setting-accelerating admixtures that accelerate cement hydration are indispensable in terms of both structural safety and economic benefits [12]. The incorporation of these activators into the system enables the manipulation of both initial and ultimate setting times while influencing the thermodynamics of the reactions, thereby directly contributing to the improvement of early-age mechanical properties [13,14].

Among the chemical admixtures used to accelerate cement hydration, CaCl_2_, an inorganic salt, is one of the oldest, most widely recognized, and most effective accelerating agents. As an accelerating admixture, CaCl_2_ increases the initial rate of the chemical reaction between cement and water, thereby promoting faster setting and hardening of concrete [12,15]. CaCl_2_ significantly shortens the reaction period by accelerating the hydration of the calcium silicate phases that constitute the principal components of cement, particularly tricalcium silicate (C_3_S) [12,14]. The increased dissolution rate of calcium ions in the system promotes the earlier and more rapid formation of C–S–H gel, which is primarily responsible for the strength development of concrete, as well as calcium hydroxide (CH) [16]. Consequently, the accelerated hydration of C_3_S results in an increase in the early-age compressive strength of concrete, particularly during the first few days after casting [10,15]. However, the use of CaCl_2_ may have adverse effects on long-term structural durability. The most critical concern is the potential for chloride ions to induce the severe corrosion of steel reinforcement in reinforced concrete structures. Owing to this corrosion risk, the use of CaCl_2_ in reinforced concrete applications has been restricted [12,17,18].

TEA is a weakly basic, polar alkanolamine widely used in the cement industry as both a grinding aid and a setting regulator [19,20,21]. The role of TEA in cement hydration and its influence on hydration kinetics vary considerably depending on its dosage [8,22]. At low dosages, TEA reacts with aluminum (Al^3+^) and iron (Fe^3+^) ions, accelerating the dissolution of the aluminate and ferrite phases in cement. This process promotes the early depletion of sulfate sources, thereby accelerating ettringite (Aft) formation and inducing the early formation of monosulfate (Afm) [9,22,23]. In contrast, at higher dosages, TEA can significantly retard the hydration of the silicate phases, either through the formation of complexes on the C_3_S surface or by inhibiting the precipitation of calcium hydroxide (CH) [20,23]. Therefore, although TEA can accelerate early setting through its effects on aluminate reactions, it may adversely affect the development of mechanical strength at later ages by suppressing silicate hydration [8,23].

The complexation capacity of TEA can disrupt the silica–alumina networks in fly ash, thereby increasing ion dissolution and accelerating the otherwise slow pozzolanic reaction. This mechanism offers considerable potential for mitigating the deficiency in early-age strength development [5,9]. Concurrently, the role of CaCl_2_ in accelerating C_3_S hydration may compensate for the well-known retarding effect of TEA on C_3_S and promote the early activation of fly ash by facilitating the more rapid release of CH into the system, which is essential for the pozzolanic reaction [14]. However, the complexity of the interactions between alkanolamines and inorganic chloride salts—including the effects of TEA on Friedel’s salt formation and the manner in which these interactions may alter the degree of polymerization of pozzolanic products, and consequently, the pore structure—makes the prediction of their synergistic effects challenging [6,11]. In the literature, studies systematically investigating the effects of the individual and combined use of CaCl_2_ and TEA on setting time, fresh-state properties, hydration behavior, and mechanical performance within the same experimental framework, particularly in cementitious systems containing fly ash, remain limited. On the other hand, minimizing cement consumption and promoting waste valorization play an important role in combating climate change. As one of the industrial wastes whose use in the concrete sector is relatively more widespread than that of other industrial wastes, fly ash slows down the hydration reactions of cementitious mixtures when used as a partial replacement for cement. In this study, the effects of increasing CaCl_2_ and TEA dosages on performance were also investigated, revealing not only whether these admixtures improve early-age performance but also their potential adverse effects on hydration development and ultimate compressive strength. Thus, the study addresses the behavior of these admixtures in fly ash-containing cementitious matrices not solely from the perspective of an “accelerating effect”, but also in terms of dosage-dependent performance variations and the relationship between hydration and mechanical properties. This comprehensive evaluation contributes to a better understanding of the limitations and potential of CaCl_2_ and TEA use in the design of more sustainable cementitious systems containing fly ash. Accordingly, this study aims to scientifically investigate how these organic and inorganic accelerating admixtures modify the setting mechanisms, pozzolanic activity kinetics, and microstructural development of fly ash-containing matrices. Furthermore, by elucidating the effects of the resulting hydration products on the early-age and ultimate mechanical performance of cementitious systems, the study aims to provide a theoretical and technological basis for the development of sustainable and high-strength composite concrete.

## 2. Experimental Program

### 2.1. Materials

**Figure 1:**
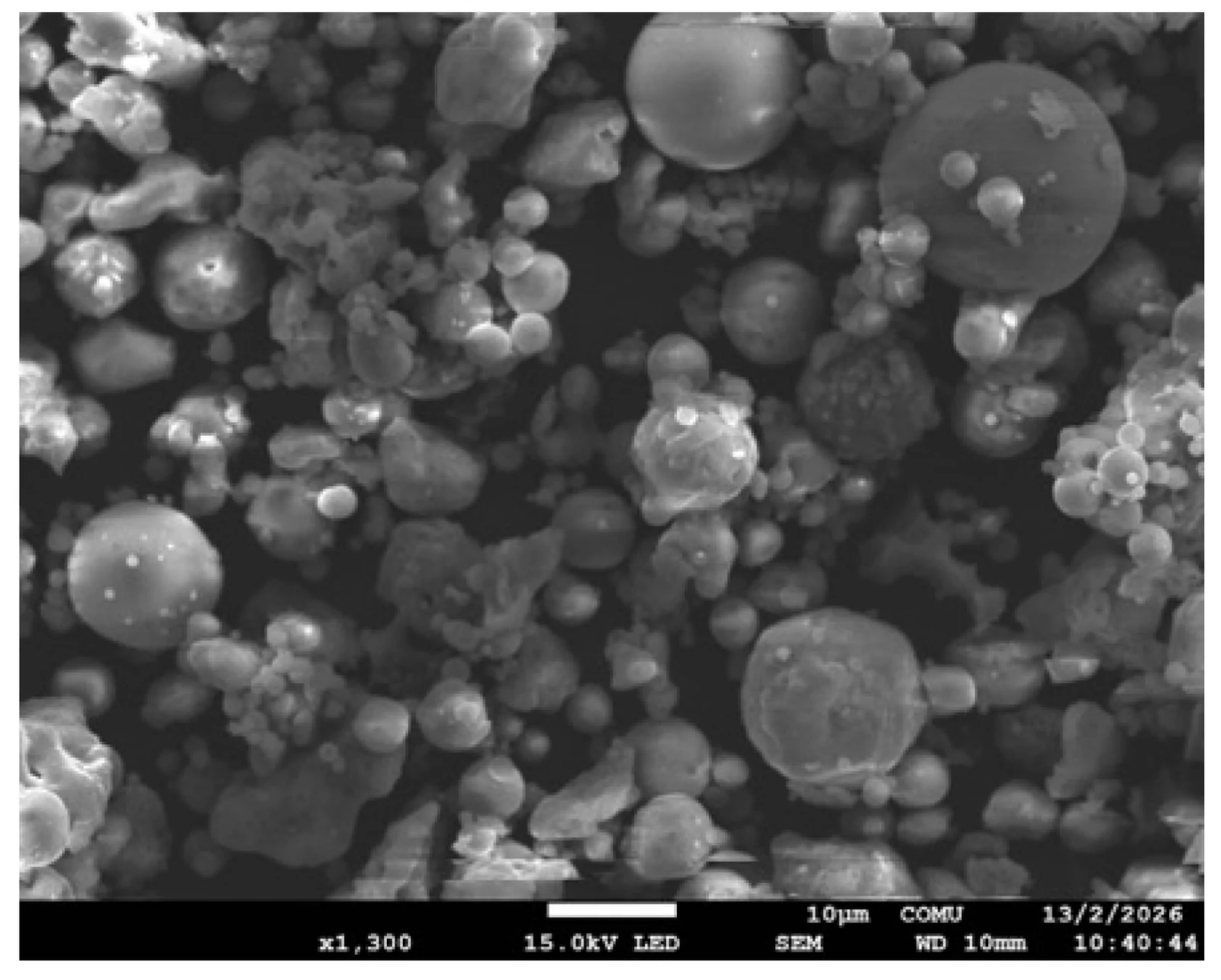
SEM image of fly ash (x1300).

In this study, CEM I 42.5R Portland cement conforming to TS EN 197-1 was used as the binder, together with Class F fly ash, which was incorporated as a 20% replacement of cement by mass. The fly ash used in this study was obtained from the Çan Thermal Power Plant, located in Çanakkale, Türkiye. The physical and chemical properties of the cement and fly ash are presented in Table 1, while the SEM image of the fly ash is shown in Figure 1. Standard CEN sand was used as the aggregate in the study. According to TS EN 1097-6, the saturated surface-dry specific gravity and water absorption capacity of the sand were determined to be 2.64 g/cm^3^ and 0.4%, respectively.

Some of the properties of the CaCl_2_ and TEA admixtures used as set accelerators, as well as the polycarboxylate ether-based water-reducing admixture (PCE) used to achieve the target flow, as obtained from the manufacturers, are presented in Table 2. The dosage of PCE was adjusted to obtain a comparable level of workability among the mortars. Specifically, 1.0 g of PCE was used for the mortars without FA, whereas 2.5 g was required for the mortars containing 20% FA. The higher PCE dosage in the FA-containing mixtures was necessary to compensate for the increased water demand and/or reduced workability associated with the incorporation of FA and to ensure that the mixtures could be prepared and tested under comparable workability conditions.

### 2.2. Mix Proportions

In the paste mixtures prepared within the scope of the study, the water-to-binder (w/b) ratio was kept constant at 0.4. For the mortar mixtures, the water-to-binder ratio, sand-to-binder ratio, and flow value were kept constant at 0.485, 2.75, and 190 ± 20 mm, respectively, in accordance with ASTM C109. In addition to the control mixture, mixtures containing 20% fly ash replacement of cement were prepared. In both the fly-ash-free and fly-ash-containing mixtures, CaCl_2_ was used as a setting accelerator at dosages of 1%, 3%, and 5% by binder weight, while TEA was used at dosages of 0.1%, 0.2%, and 0.3%. In addition, PCE was used in all mortar mixtures to achieve the target flow value. The quantities of materials used in all paste and mortar mixtures are presented in Table 3 and Table 4 respectively. The mixtures were designated based on the contents of fly ash, CaCl_2_, and TEA. For example, the control mixture was coded as C; the mixture containing 1% CaCl_2_ and no fly ash was coded as CC1; and the mixture containing fly ash, 3% CaCl_2_, and 0.3% TEA was coded as FA-CC3-TEA03.

### 2.3. Test Methods

**Figure 2:**
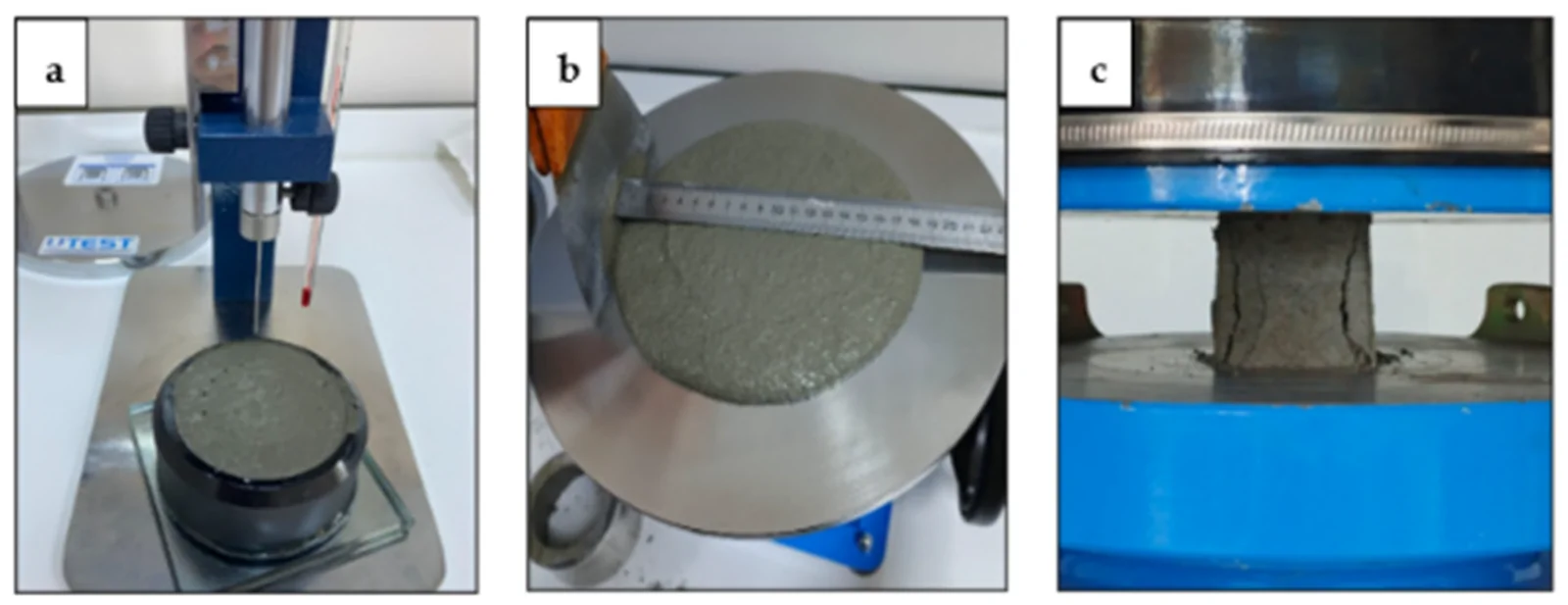
Images of the experiments: (**a**) Vicat test, (**b**) time-dependent flow, and (**c**) compression test.

The Vicat test was conducted to determine the setting times of the paste and mortar mixtures, while a time-dependent flow spread test was performed to evaluate their consistency retention performance. In this context, 20 paste mixtures were prepared for the Vicat test, and 20 mortar mixtures were prepared for the time-dependent flow spread test. The Vicat test was conducted in accordance with EN 196-3 (Figure 2a). The same water-to-binder ratio was maintained for all paste mixtures to ensure consistency in the experimental procedure and to investigate the effects of the incorporated materials. The resulting consistency values were considered acceptable when they fell within the lower and upper limits specified by the applicable standard requirements. However, the use of a constant water-to-binder ratio does not necessarily ensure identical consistency among mixtures with different compositions. The flow spread values were measured at 0, 20, 40, and 60 min in accordance with ASTM C1437 (Figure 2b). A total of 360 cubic specimens measuring 50 × 50 × 50 mm were produced to determine the compressive strength and water absorption capacity of the mixtures. The mixtures were prepared in accordance with ASTM C109, and following placement into the molds, were kept in the molds for 24 h at a temperature of 22 ± 2 °C and a relative humidity of 95%. After this period, the specimens were demolded and cured in lime-saturated water at 22 ± 2 °C until the corresponding testing ages. Compressive strength values at 1, 3, 7, and 28 days were determined in accordance with ASTM C109 (Figure 2c), while water absorption values at 7 and 28 days were determined in accordance with ASTM C642.

### 2.4. Statistical Analysis

All mechanical test results were expressed as mean values obtained from at least three specimens for each data point. At least three specimens were used for the tests. Setting time measurements were evaluated based on both the initial and final setting times, while the flowability tests were assessed based on the initial flow diameter and the changes in flow diameter over time.

To assess the statistical significance of the differences among the mixtures, a one-way ANOVA was performed at a 95% confidence level (*p* < 0.05). Due to the limited number of replicates (*n* = 3), the ANOVA results were interpreted primarily as supporting evidence for the observed performance differences and trends among the mixtures, rather than as definitive inferential conclusions. The compressive strength, setting time, water absorption, flowability, and time-dependent flowability results were evaluated collectively to identify differences in the mechanical performance, workability, and time-dependent behavior of the mixtures.

## 3. Results and Discussion

### 3.1. Setting Time

**Figure 3:**
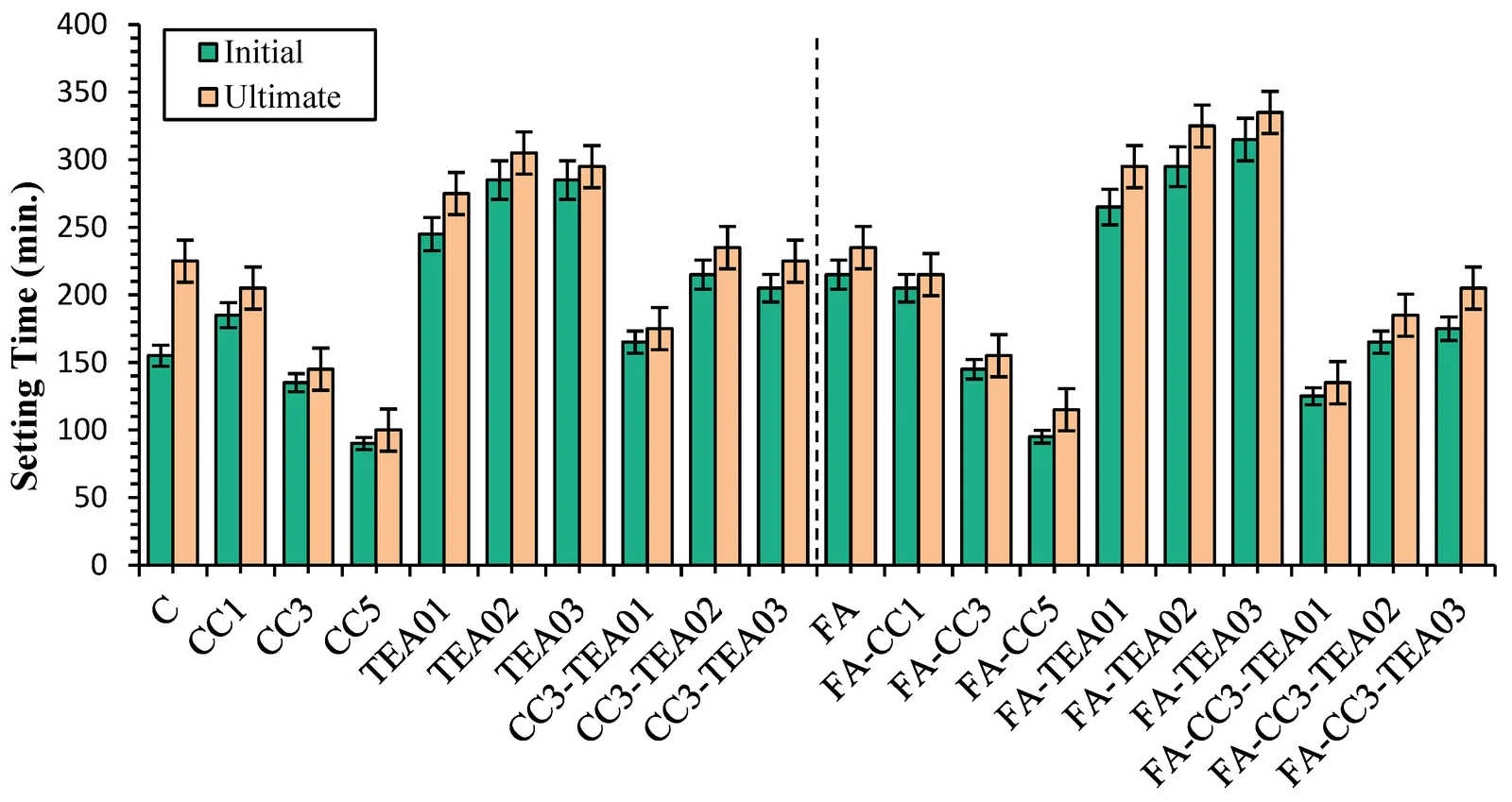
Initial and ultimate setting times of the mixtures.

Figure 3 presents the initial and ultimate setting times of the cement paste mixtures. Setting time is one of the important macroscopic indicators of hydration reactions in cement-based systems and is a key parameter that directly affects the workability period, placement, and early-age mechanical behavior of fresh materials. The occurrence of initial setting indicates the onset of the transition from a plastic to a rigid state, whereas ultimate setting indicates that this transition has been largely completed [24].

In the control mixture, the initial and ultimate setting times were measured as 155 and 225 min, respectively. For the FA mixture containing fly ash, the initial and ultimate setting times were approximately 215 and 235 min, respectively. The approximately 60-min increase in the initial setting time compared with the control mixture indicates that the incorporation of fly ash delayed the setting process. This behavior can be attributed to the lower initial rate of hydration resulting from the reduced amount of Portland cement participating in the hydration reactions when fly ash is used as a partial replacement for Portland cement. Furthermore, the extent of this delay may also depend on the class and characteristics of the fly ash [25,26].

As shown in Figure 3, the mixtures containing CaCl_2_ also exhibited a pronounced reduction in setting time compared with the control mixture, depending on the dosage used. Although the initial setting time, which was approximately 155 min for the control mixture, increased slightly to approximately 185 min with the incorporation of 1% CaCl_2_, it decreased to approximately 135 and 90 min for the mixtures containing 3% and 5% CaCl_2_, respectively. Similarly, while the ultimate setting time was approximately 225 min for the control mixture, it decreased to approximately 205, 145, and 100 min for the mixtures containing 1%, 3%, and 5% CaCl_2_, respectively. These results indicate that CaCl_2_ promotes earlier solidification by accelerating hydration in cementitious systems [27,28]. The increased ionic concentration in the system resulting from the dissolution of CaCl_2_ accelerates the reactions of the aluminate phases, particularly tricalcium aluminate (C_3_A), leading to greater heat evolution during the early stages of hydration. This accelerated reaction kinetics contributes to shortening the setting time of cement and enables the hydration heat flow to reach its maximum value earlier and at a higher magnitude. The effect of CaCl_2_ is not limited to C_3_A hydration; it also accelerates the hydration of tricalcium silicate (C_3_S), thereby promoting the formation of calcium silicate hydrate (C–S–H) gel and calcium hydroxide (CH), which are the primary hydration products at early ages. Consequently, the incorporation of CaCl_2_ alters the reaction kinetics of both aluminate and silicate phases, resulting in accelerated hydration and enabling the cementitious system to develop a rigid structure within a shorter period [14,29].

For the mixtures containing fly ash and CaCl_2_ at dosages of 1%, 3%, and 5%, the initial setting times were approximately 205, 145, and 95 min, respectively, while the corresponding ultimate setting times were approximately 215, 155, and 105 min. These results indicate that CaCl_2_ exhibited its set-accelerating effect in the fly ash-containing mixtures, similar to that observed in the fly ash-free mixtures, thereby mitigating the setting delay caused by the incorporation of fly ash. In particular, the initial setting time of the FA mixture, which was approximately 215 min, decreased to approximately 95 min when 5% CaCl_2_ was used. Thus, the incorporation of 5% CaCl_2_ resulted in a reduction of approximately 120 min in the initial setting time.

When the mixtures containing TEA are considered, a pronounced setting-retarding tendency is observed, in contrast to the CaCl_2_ series. For the mixtures containing TEA at dosages of 0.1%, 0.2%, and 0.3%, the initial setting times were approximately 245, 285, and 285 min, respectively, while the corresponding ultimate setting times were approximately 275, 305, and 295 min. Compared with the control mixture, the initial setting time was found to increase by approximately 130 min, particularly in the mixtures containing 0.2% and 0.3% TEA. The effect of TEA on cement setting cannot simply be characterized as either retarding or accelerating. Previous studies have reported that TEA can produce considerably different setting behaviors depending on its dosage, cement composition, particularly the C_3_A content, sulfate balance, and the presence of other admixtures. For instance, TEA may accelerate setting at low or appropriate dosages; however, as the dosage increases, its effect on setting behavior may become more complex [11,22]. At low dosages (e.g., 0.02%), TEA undergoes complexation reactions with Ca^2+^, Al^3+^, and Fe^3+^ ions in the pore solution and accelerates the dissolution of the C_3_A and C_4_AF phases. As gypsum in the solution is consumed at an earlier stage, ettringite formation is accelerated, followed by its conversion to monosulfate, resulting in rapid stiffening of the cement paste [9,23]. At higher dosages, however, TEA may retard the hydration of calcium silicates. TEA–calcium complexes can accumulate on the surface of C_3_S particles, forming a low-permeability barrier layer that limits contact between water and the silicate particles and consequently restricts C–S–H gel formation. In addition, TEA may retard setting by inhibiting the crystallization of calcium hydroxide (CH) [5,23].

The combined use of fly ash and TEA resulted in a pronounced increase in setting time compared with all other mixtures. In particular, the mixture containing 0.3% TEA exhibited the longest initial setting time among all mixtures, reaching approximately 315 min. The ultimate setting time also reached a maximum value of approximately 335 min. The substantial increase in setting time can be attributed to the combined retarding effects of both fly ash and TEA. For the mixtures in which 3% CaCl_2_ was used in combination with 0.1%, 0.2%, and 0.3% TEA, the initial setting times were approximately 165, 215, and 205 min, respectively, while the corresponding ultimate setting times were approximately 175, 235, and 225 min. The addition of CaCl_2_ to the TEA-containing mixtures resulted in a pronounced reduction in setting times, demonstrating the effective set-accelerating action of CaCl_2_ in mitigating the retarding effect of TEA.

### 3.2. Time-Dependent Flow

**Figure 4:**
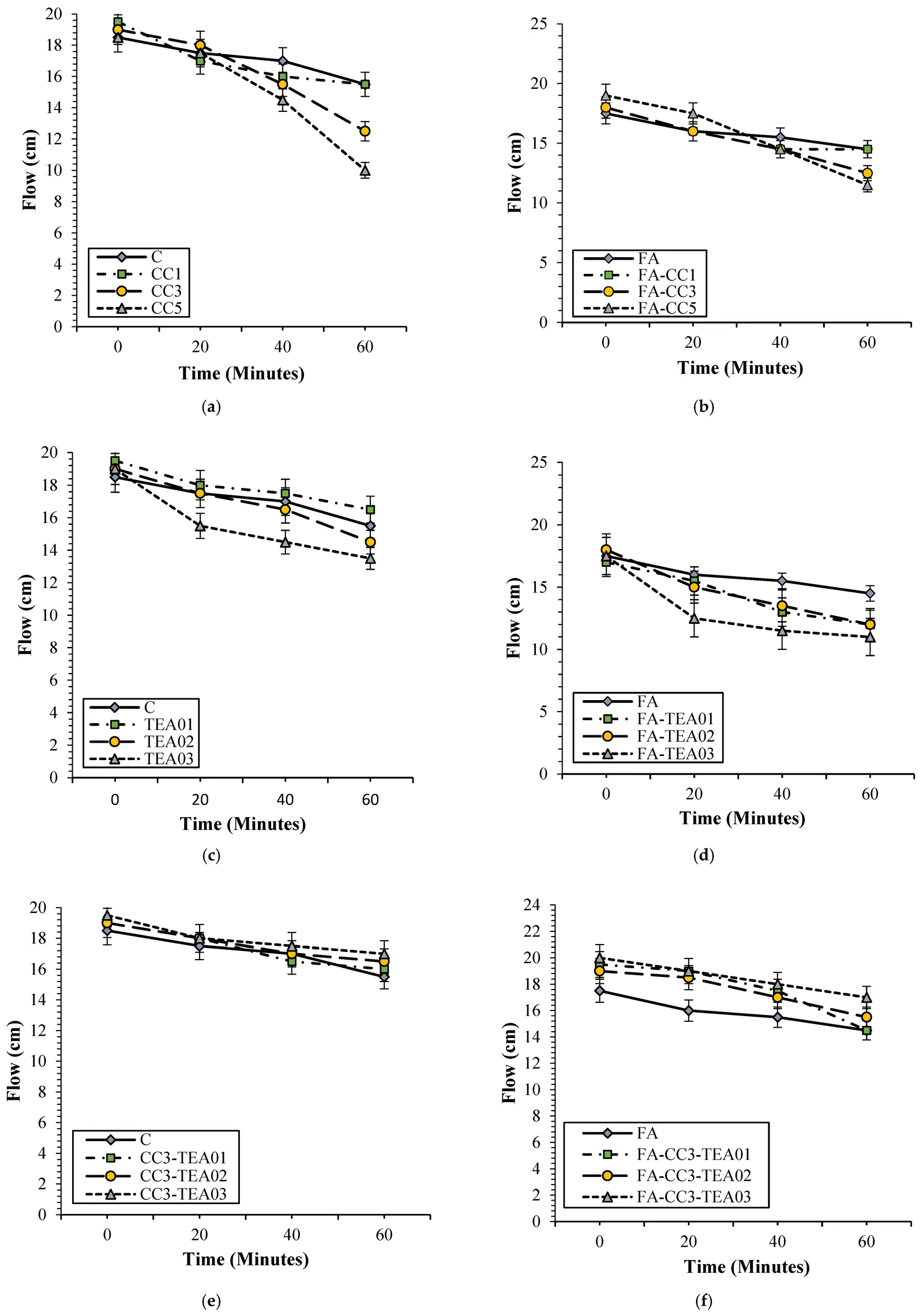
Flow values of the mixtures between 0 and 60 min.

Figure 4 presents the time-dependent spread values of the mixtures. The initial spread value of the control mixture was 18.5 cm, which decreased to 15.5 cm at 60 min. The addition of CaCl_2_ to the control mixture resulted in a reduction in the time-dependent spread. The mixture containing 1% CaCl_2_ exhibited a relatively greater loss of consistency compared with the control mixture. For the mixtures containing 3% and 5% CaCl_2_, the spread values at 60 min decreased markedly. The mixture incorporating 5% CaCl_2_ exhibited an approximately 45% loss of consistency relative to its initial value. This dosage-dependent loss of consistency can be attributed directly to the effect of CaCl_2_ on the early hydration of cement. It is well-established that the Cl^−^ ions released upon dissolution of CaCl_2_ accelerate early ettringite formation, while Ca^2+^ ions promote the nucleation of C–S–H phases [30,31].

When the effect of CaCl_2_ on all mixtures containing fly ash was examined, it was found that the overall trend was consistent with that observed for the mixtures without fly ash. The mixture containing fly ash and 5% CaCl_2_ exhibited a spread value of approximately 12 cm at 60 min, whereas the mixture without fly ash and containing 5% CaCl_2_ showed no measurable spread, reaching a value of approximately 10 cm under the same conditions. The markedly slower pozzolanic reactivity of fly ash compared with Portland cement does not result in significant chemical water consumption during the early stages. Consequently, even at a high CaCl_2_ dosage, the presence of fly ash contributes to the relative preservation of fresh-state consistency [32].

When the effects of TEA on the mixtures without fly ash were evaluated, a different behavior was observed compared with the CaCl_2_ series. The mixtures containing 0.1% and 0.2% TEA initially exhibited spread values close to that of the control mixture, and this behavior was maintained up to 60 min. Lu et al. [33] experimentally demonstrated that at low dosages, TEA weakens the electrostatic attractive forces between cement particles, thereby reducing the yield stress of fresh mortar. However, the same study reported that TEA dosages exceeding 0.2% can promote the acceleration of aluminate-containing phases, resulting in rapid setting behavior. This finding is consistent with the low spread value observed for the mixture containing 0.3% TEA. Furthermore, the acceleration of ettringite and monosulfate phase formation induced by TEA contributes to an increase in the loss of consistency [33].

In the mixtures containing both TEA and fly ash, the initial spread values were found to be close to that of the FA control mixture as the TEA dosage increased; however, the spread values measured at 60 min remained markedly lower than that of the control mixture. Lu et al. [21] demonstrated that TEA can temporarily alter the active ionic species involved during ettringite formation. Given its high content of aluminum- and silicon-rich phases, fly ash is considered to interact with TEA molecules, thereby contributing to an increased rate of consistency loss.

In all mixtures in which CaCl_2_ and TEA were used in combination, spread values higher than those of the control mixture were observed both initially and at 60 min. When CaCl_2_ was used alone at a dosage of 3%, the initial spread value of approximately 19 cm decreased to 12 cm at 60 min. In contrast, for the mixtures containing both CaCl_2_ and TEA, the initial spread value of 19 cm decreased to approximately 16–17 cm at 60 min, depending on the TEA dosage. This finding indicates that the addition of TEA significantly retarded the effect of CaCl_2_ on consistency loss. In the study conducted by Chen Jixi et al. [11], it was reported that the addition of CaCl_2_ to the mixtures facilitates the dispersion of agglomerated large particles, allowing excess water to surround the cement particles and increasing the thickness of the water film. The increased water-film thickness was observed to improve the contact area between the cement particles and water, thereby contributing to cement hydration. Furthermore, the addition of TEA to the mixtures was shown to promote the formation of complexes with Ca^2+^ in the cement clinker, resulting in the adsorption of TEA–Ca complexes onto the surfaces of cement particles. It was reported that the hydration of these complex-coated cement particles became more difficult, which subsequently had an adverse effect on strength development. Based on these findings, it is considered that in mixtures containing both CaCl_2_ and TEA, the increase in water-film thickness induced by CaCl_2_, together with the formation of TEA–Ca complexes on the surfaces of cement particles induced by TEA, limits the availability of the water film for hydration. Consequently, a greater amount of water remains in the free state, thereby extending the time required for consistency loss.

In the mixtures containing fly ash in which CaCl_2_ and TEA were used in combination, the spread values were higher than that of the FA control mixture. These mixtures maintained their relatively high initial spread values, approximately 19–20 cm, well up to 60 min. The reduced hydration rate resulting from the combined use of CaCl_2_ and TEA may suppress the pozzolanic reactivity of fly ash during the early stages, thereby contributing to the retention of higher spread values.

**Figure 5:**
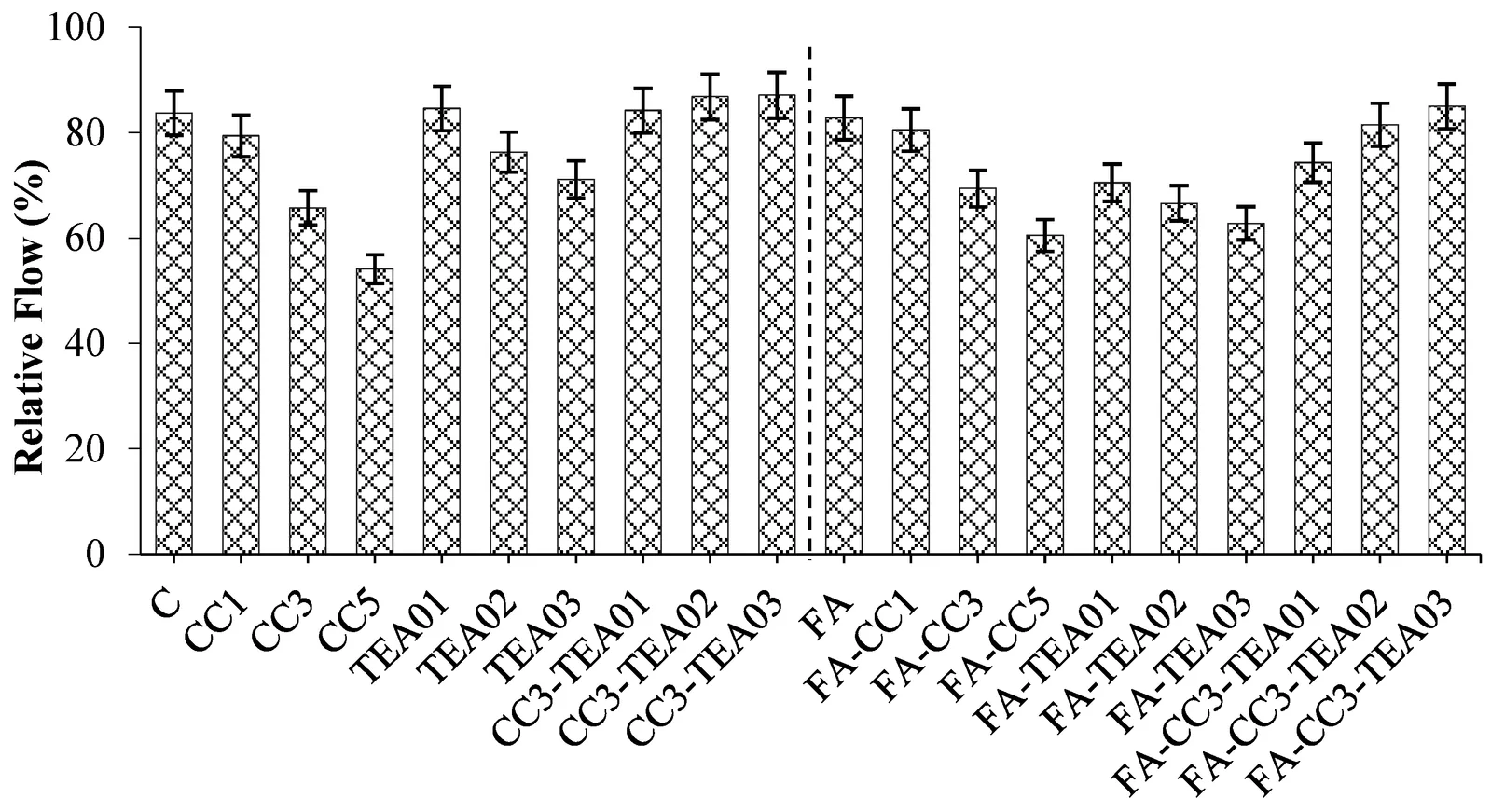
Relative rates of time-dependent diffusion in mixtures.

Figure 5 presents the relative rates of time-dependent spread in the mixtures. Overall, the results indicate that the rate and magnitude of slump loss varied depending on the type and dosage of the admixture. CaCl_2_ shortened the workability period, particularly at higher dosages, in mixtures without fly ash. TEA exhibited a dosage-dependent and variable effect when used alone, whereas its combination with CaCl_2_ significantly slowed the rate of slump loss. Fly ash replacement extended the workability period, particularly when used in combination with CaCl_2_ and TEA.

### 3.3. Compressive Strength

**Figure 6:**
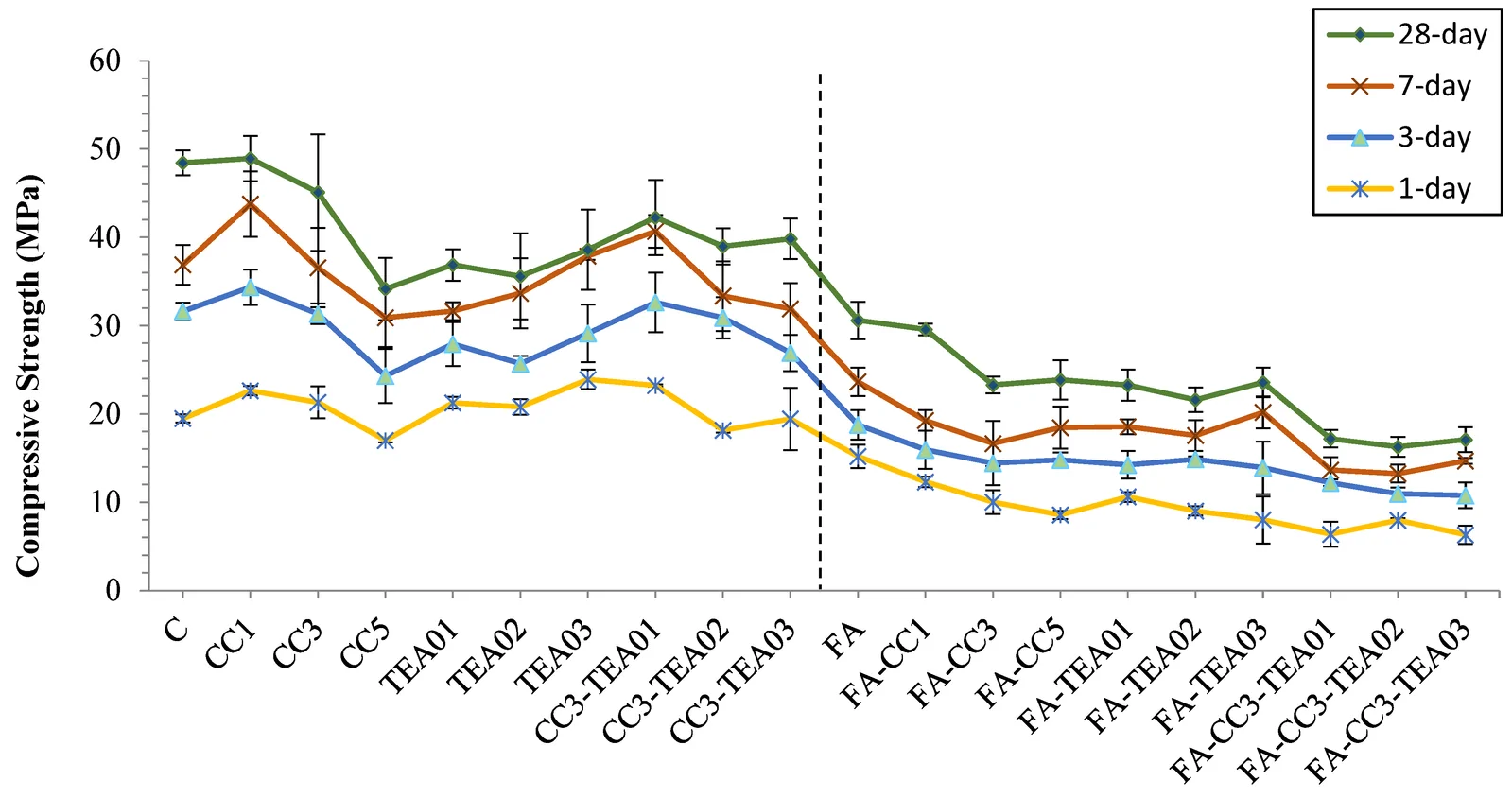
Compressive strength of mixtures.

**Figure 7:**
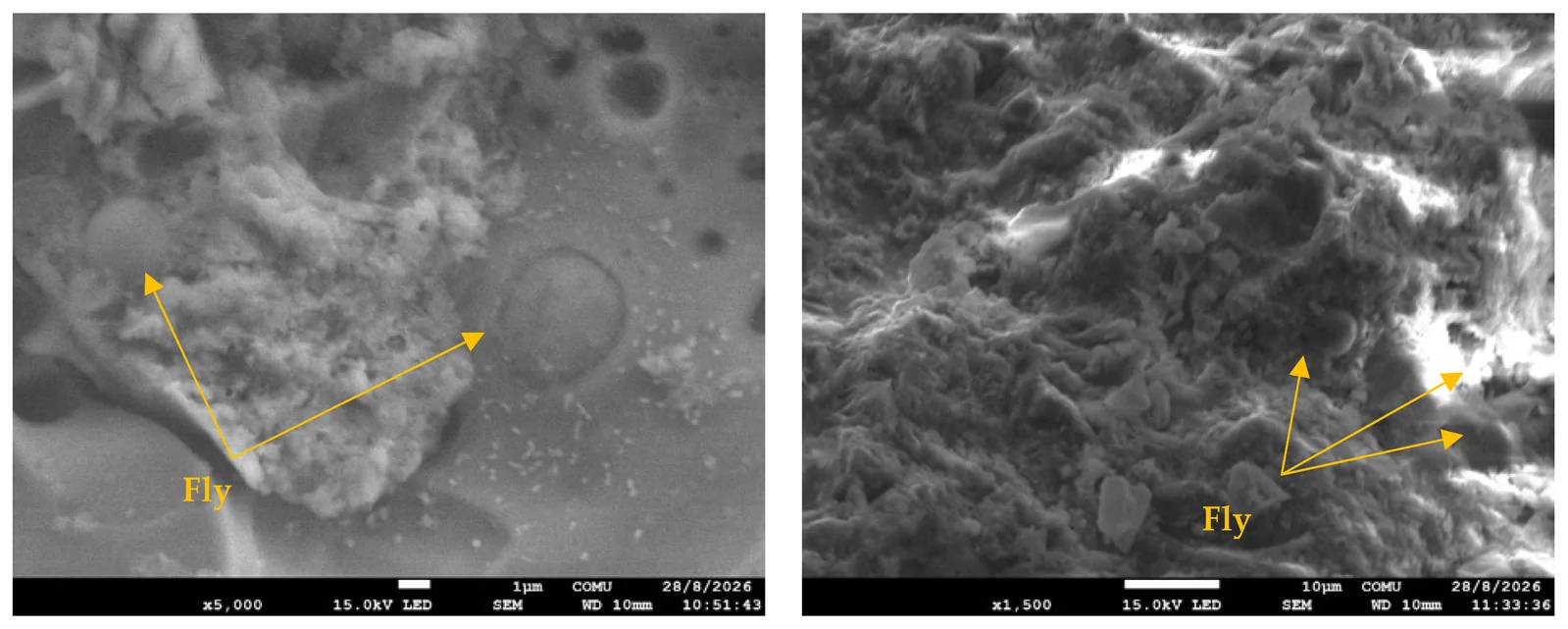
SEM images of unreacted fly ash.

The 1-, 3-, 7-, and 28-day compressive strengths of the mixtures containing fly ash, CaCl_2_, and TEA are presented in Figure 6. Each value was determined by calculating the average of three specimens. The 1-day compressive strengths of the control and FA specimens containing fly ash were 19.47 and 15.19 MPa, respectively, while the corresponding 28-day compressive strengths were 48.43 and 30.59 MPa, respectively. These results indicate that replacing 20% of the cement with fly ash resulted in an approximately 22% reduction in early-age compressive strength and an approximately 36% reduction in later-age compressive strength. This decrease in strength is considered to be attributable to the type of fly ash used. In the study conducted by Oner et al. [34], it was emphasized that increasing the cement content leads to an increase in the amount of Ca(OH)_2_ formed as a result of hydration. Consequently, the amount of Ca(OH)_2_ available for consumption by fly ash during pozzolanic reactions increases, resulting in greater formation of C–S–H (calcium silicate hydrate). The authors reported that after reaching a certain maximum strength, a subsequent decrease in strength was associated with the presence of excessive amounts of unreacted fly ash in the system. Unreacted fly ash particles were stated to act as fine aggregate-like constituents within the microstructure, with their contribution to the mechanical properties being limited primarily to a filler effect. The SEM images obtained in the present study, shown in Figure 7, also indicate that unreacted fly ash particles are present within the matrix, where they function primarily as filler material.

According to the test results, the addition of CaCl_2_ to the mixtures without fly ash resulted in different compressive strength values depending on the dosage. When CaCl_2_ was used at a dosage of 1%, the 1-day early-age compressive strength increased by 16% compared with the control mixture, while the 28-day compressive strength increased by approximately 1%. At a CaCl_2_ dosage of 3%, the 1-day compressive strength increased by 9.39% relative to the control mixture, whereas the 28-day compressive strength decreased by 6.92%. CaCl_2_ affects the hydration of C_3_S and C_3_A, thereby promoting rapid setting and increasing early-age strength. When the CaCl_2_ dosage was increased to 5%, the 1-day and 28-day compressive strengths decreased by 12.71% and 29.48%, respectively. Thus, increasing the CaCl_2_ dosage adversely affected both early-age and later-age compressive strengths. Previous studies have suggested that a high concentration of CaCl_2_ may lead to the formation of an impermeable hydration layer on the surface of cement particles at an early stage, thereby restricting the diffusion of water and ions. In addition, the modifying effects of high chloride concentrations on ettringite morphology may contribute to long-term strength losses [27,28].

**Figure 8:**
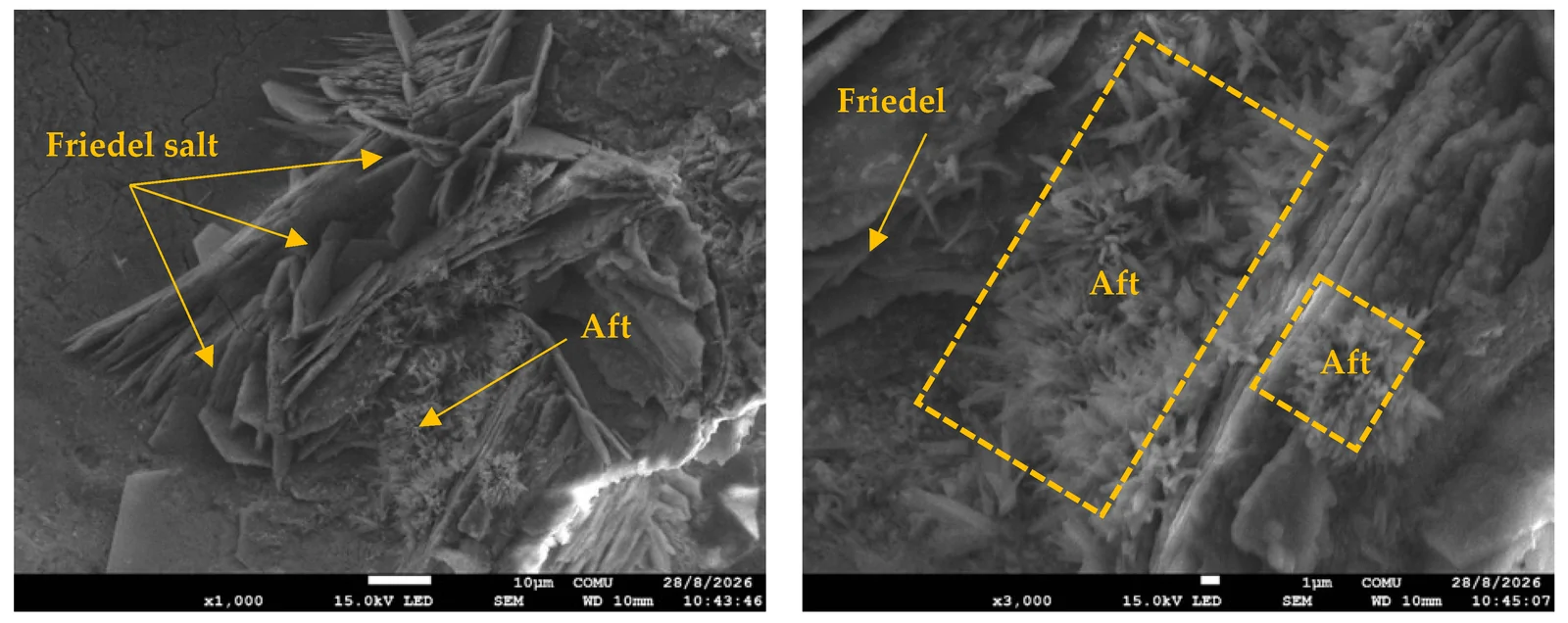
Images of ettringite and Friedel’s salt formations.

The literature indicates that the use of CaCl_2_ leads to high early-age strength in Portland cement concrete; however, the increase in compressive strength at later ages is not significant. It has also been reported that the addition of CaCl_2_ at dosages ranging from 1% to 5% of the binder weight does not significantly affect compressive strength, whereas the use of CaCl_2_ at dosages above 5% results in a reduction in compressive strength [35]. In the present study, the decrease in strength with increasing CaCl_2_ content is considered to be attributable to the accelerated setting, which may prevent the hydration process from proceeding to completion in a uniform manner. The SEM images presented in Figure 8 show the formation of ettringite and Friedel’s salt. CaCl_2_ affects the hydration of C_3_S, C_3_A, and gypsum. CaCl_2_ increases the dissolution rate of gypsum, thereby increasing the amount of SO_4_^2−^ in the solution and accelerating ettringite formation. Depending on the Cl^−^ concentration in the solution, Kuzel’s salt or Friedel’s salt may form. In addition, owing to their small ionic size, Cl^−^ ions can penetrate the protective C–S–H layer, leading to its deterioration [14].

TEA is an admixture used in concrete production to counteract the setting retardation caused by chemical admixtures, thereby enhancing early-age strength, and to accelerate the pozzolanic reactions of mineral admixtures such as fly ash, thereby improving early-age performance [20].

When TEA was added to the mixtures without fly ash, a different behavior from that observed with CaCl_2_ was encountered. The test results showed that the 1-day compressive strengths of the TEA-containing mixtures increased compared with that of the control mixture; however, a decrease in later-age strength was observed from the 3rd day onward. The 1-day compressive strengths of the mixtures containing 0.1%, 0.2%, and 0.3% TEA increased by 9.17%, 6.80%, and 22.73%, respectively, compared with the control mixture, whereas the corresponding 28-day compressive strengths decreased by 23.86%, 26.51%, and 20.29%, respectively. This behavior may be attributed to the formation of a dense layer around the cement particles as a result of rapid hydration at early ages, which may delay subsequent-age hydration, as well as to the formation of a porous microstructure due to the development of denser hydration products [30]. At low dosages (0.02–0.05%), TEA acts as a setting accelerator, thereby increasing early-age compressive strength. At moderate dosages (0.1–0.5%), its retarding effect on hydration becomes dominant, and reductions in later-age strength may be observed. At high dosages (1.0% and above), TEA again exhibits an accelerating effect, leading to flash setting; however, due to adverse changes in the microstructure, the later-age compressive strength decreases considerably [20,23].

According to the test results obtained for the series in which CaCl_2_ and TEA were used in combination, the mixture containing 3% CaCl_2_ and 0.1% TEA exhibited a compressive strength of 23.22 MPa at day 1, which was 19% higher than that of the control mixture. At 7 days, the compressive strength reached 40.67 MPa, corresponding to a value 10% higher than that of the control mixture. The early-age hydration effects of both admixtures can be considered responsible for this increase in strength. However, at 28 days, the compressive strength decreased by approximately 12.78% compared with that of the control mixture. When the TEA content was increased to 0.2% and 0.3%, reductions of 11–30% were observed in both the early- and later-age compressive strength values of the mixtures. This finding indicates that establishing an appropriate balance between the dosages of CaCl_2_ and TEA is of critical importance when these admixtures are used in combination.

Under normal conditions, fly ash reacts pozzolanically with calcium hydroxide (CH), which is released as a by-product of Portland cement hydration, forming calcium silicate hydrate (C–S–H) gels that contribute to strength development [34]. The addition of CaCl_2_ to the mixture is expected to accelerate this pozzolanic reaction process, thereby enabling fly ash to also contribute to the system at early ages [3]. However, contrary to this expectation, in the present study, the incorporation of CaCl_2_ into fly ash-containing mixtures resulted in a pronounced reduction in both early- and later-age compressive strengths compared with the control mixture (FA). The 1-day compressive strengths of the mixtures containing fly ash and 1%, 3%, and 5% CaCl_2_ decreased by 18.99%, 34.14%, and 43.62%, respectively, while the corresponding 28-day compressive strengths decreased by 3.36%, 23.91%, and 22.03%, respectively. The mechanism underlying this adverse interaction can be explained by the chemical dynamics associated with the specific requirements of the pozzolanic reaction of fly ash. Chloride ions present in the hydration environment can react with aluminate phases in cement hydration products, leading to the formation of Friedel’s salt and related chloroaluminate hydrates. During this process, the consumption of a certain amount of Ca(OH)_2_ reduces the availability of this essential reactant for the pozzolanic reaction of fly ash, thereby affecting the formation of secondary C–S–H [36,37].

A similar adverse effect was observed in the mixtures containing TEA and fly ash. The compressive strength of all mixtures remained below that of the FA control mixture. In the mixtures containing 0.1%, 0.2%, and 0.3% TEA, the 1-day compressive strengths decreased by 30.26%, 40.71%, and 47.33%, respectively, compared with the control mixture, while the corresponding 28-day compressive strengths decreased by 23.97%, 29.42%, and 22.93%, respectively.

The mixture group exhibiting the lowest compressive strength values in the study consisted of the series in which 3% CaCl_2_, TEA, and fly ash were used simultaneously. The 1-day compressive strengths of the mixtures containing 3% CaCl_2_ and 0.1%, 0.2%, and 0.3% TEA decreased by 58.04%, 47.60%, and 58.41%, respectively, compared with the control mixture. At 28 days, the corresponding decreases were 43.83%, 46.79%, and 44.12%, respectively. This loss in strength can be explained by the simultaneous occurrence of Ca(OH)_2_ consumption induced by CaCl_2_ and the potential passivation of reactive surfaces by TEA, which may have restricted the initiation of the pozzolanic reaction. In addition to the adverse effects of CaCl_2_ and TEA when used independently, their combined use appears to have intensified these negative effects in a simultaneous and cumulative manner [8,14,21,30].

Taken as a whole, these findings indicate that the effectiveness of the investigated admixtures is largely dependent on the binder composition and dosage level. Among the admixtures investigated, low-dose CaCl_2_ (1%) in mixtures without fly ash stands out as the only admixture condition that exceeded the reference values in terms of both early- and later-age compressive strength. Mixtures containing fly ash exhibited systematic and multifaceted sensitivity to both admixtures and their combined use. Rather than promoting pozzolanic activation, the presence of the admixtures appears to have exerted an inhibitory effect under all conditions investigated. These findings demonstrate that the selection of chemical admixtures for fly ash-blended mixtures requires an independent and specific evaluation framework rather than one directly derived from Portland cement-based systems. Furthermore, in future studies, the interaction mechanisms between pozzolanic and chemical admixtures should be investigated in greater depth through comprehensive experimental studies.

**Figure 9:**
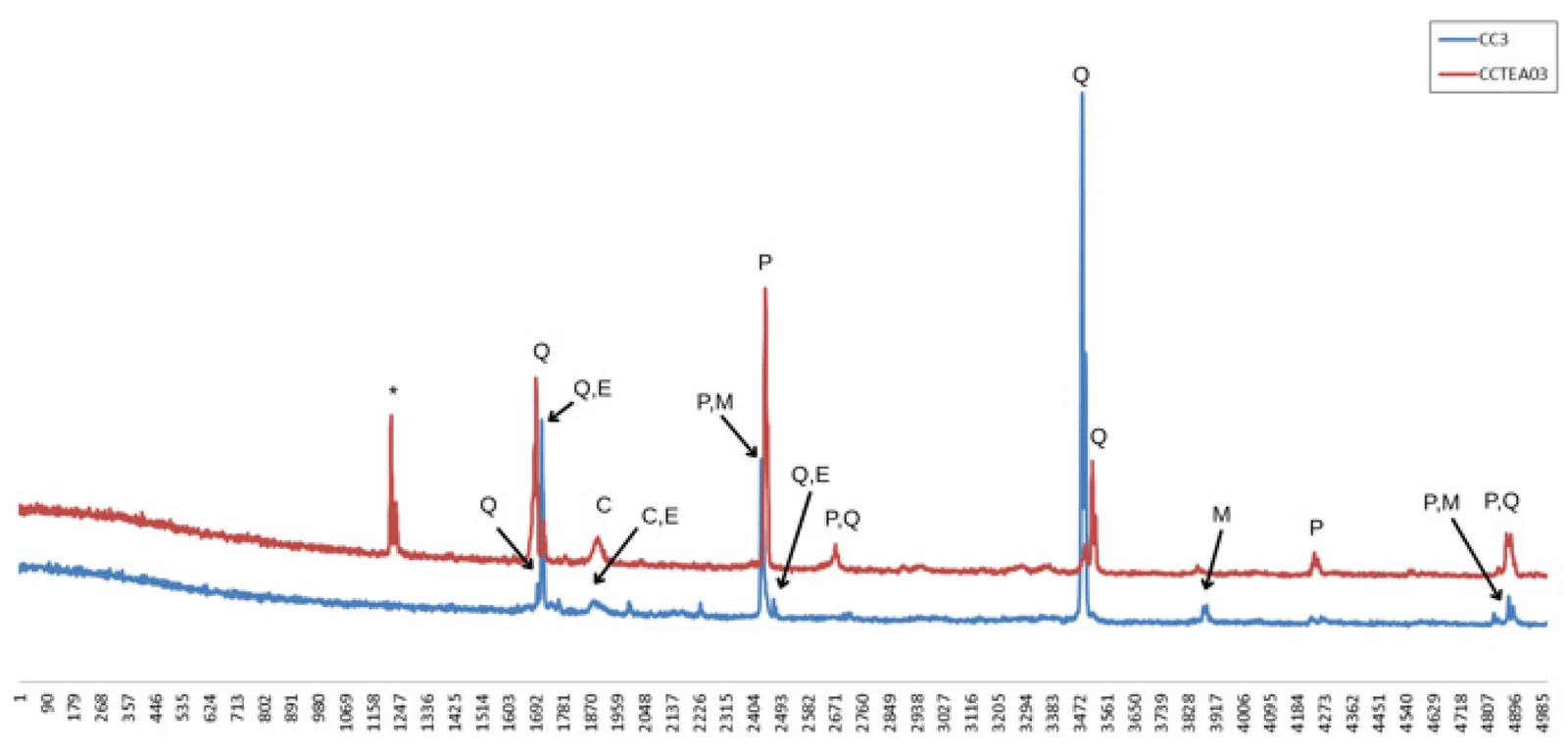
XRD patterns of the mortar specimens: CC3 and CCTEA03.

On the other hand, the XRD analysis results obtained from mortar specimens prepared from the CC3 and CCTEA03 mixtures are presented in Figure 9. As shown in Figure 9, the dominant phases were identified as quartz (2θ ≈ 26.96° and 50.2°), portlandite (2θ ≈ 36.6° and 60.1°), and calcite (2θ ≈ 29.4°). Due to the pronounced high-intensity quartz reflections originating from the aggregate in the mortar specimens, minor hydration products such as AFm and AFt phases could not be reliably identified by XRD analysis. Therefore, the XRD results obtained in this study were primarily evaluated in terms of identifying the dominant crystalline phases present in the specimens and assessing the differences observed in the TEA-containing specimens. In Figure 9, an unidentified reflection at approximately 20.8° 2θ was observed exclusively in the TEA-containing specimens. Since TEA is known to accelerate C_3_A hydration, the possibility that this reflection originates from residual C_3_A appears unlikely. However, the available XRD results do not allow for the definitive identification of this peak. Although the reflection may be associated with a TEA-related intermediate phase or a modified hydration product, this interpretation requires further confirmation using complementary analytical techniques such as TGA, DSC, or solid-state NMR.

Since the XRD analyses in this study were performed on mortar specimens and the primary scope of the study was to evaluate the overall properties of the mortars rather than to elucidate the detailed hydration mechanisms of the admixtures, no definitive mechanism regarding the effect of TEA on the hydration process is proposed. Nevertheless, the XRD findings suggest that the incorporation of TEA may influence the formation of hydration products. In future studies, the effects of TEA and other admixtures on hydration mechanisms will be investigated in greater detail using XRD analyses of cement paste specimens in conjunction with complementary techniques such as TGA, solid-state NMR, and other advanced characterization methods. In addition, the effects of these admixtures on the long-term performance and durability properties of mortars under different environmental exposure conditions will be systematically evaluated.

### 3.4. Water Absorption

**Figure 10:**
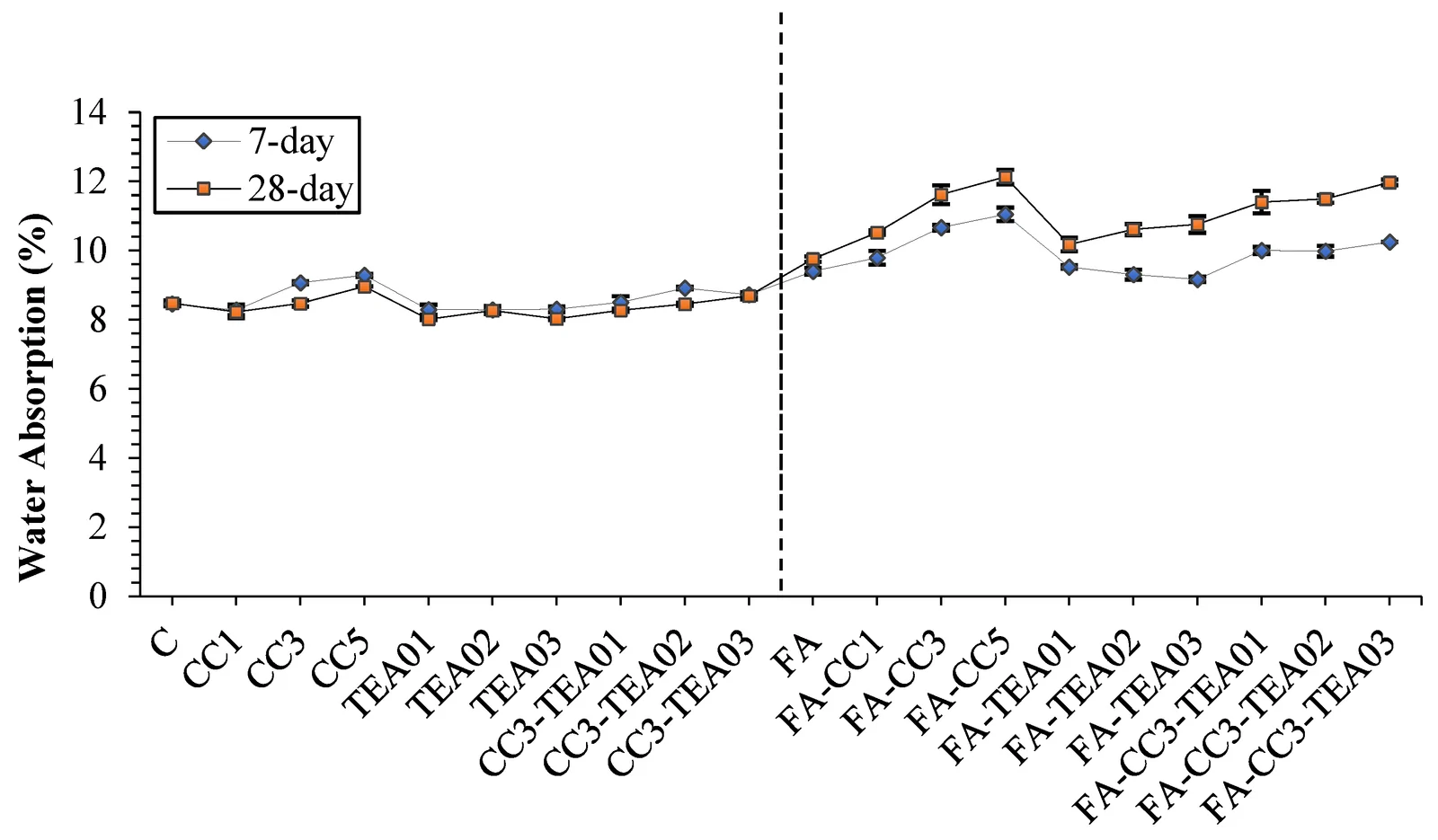
Water absorption values of the mixtures.

The 7- and 28-day water absorption rates of the mixtures containing fly ash, CaCl_2_, and TEA are presented in Figure 10. Each value was determined by calculating the average of three specimens. Water absorption rate is an important parameter that represents the amount of water absorbed into the internal structure of concrete specimens. This parameter is influenced by various factors and is considered one of the indicators that enables the prediction of the properties of concrete mixtures [38].

**Figure 11:**
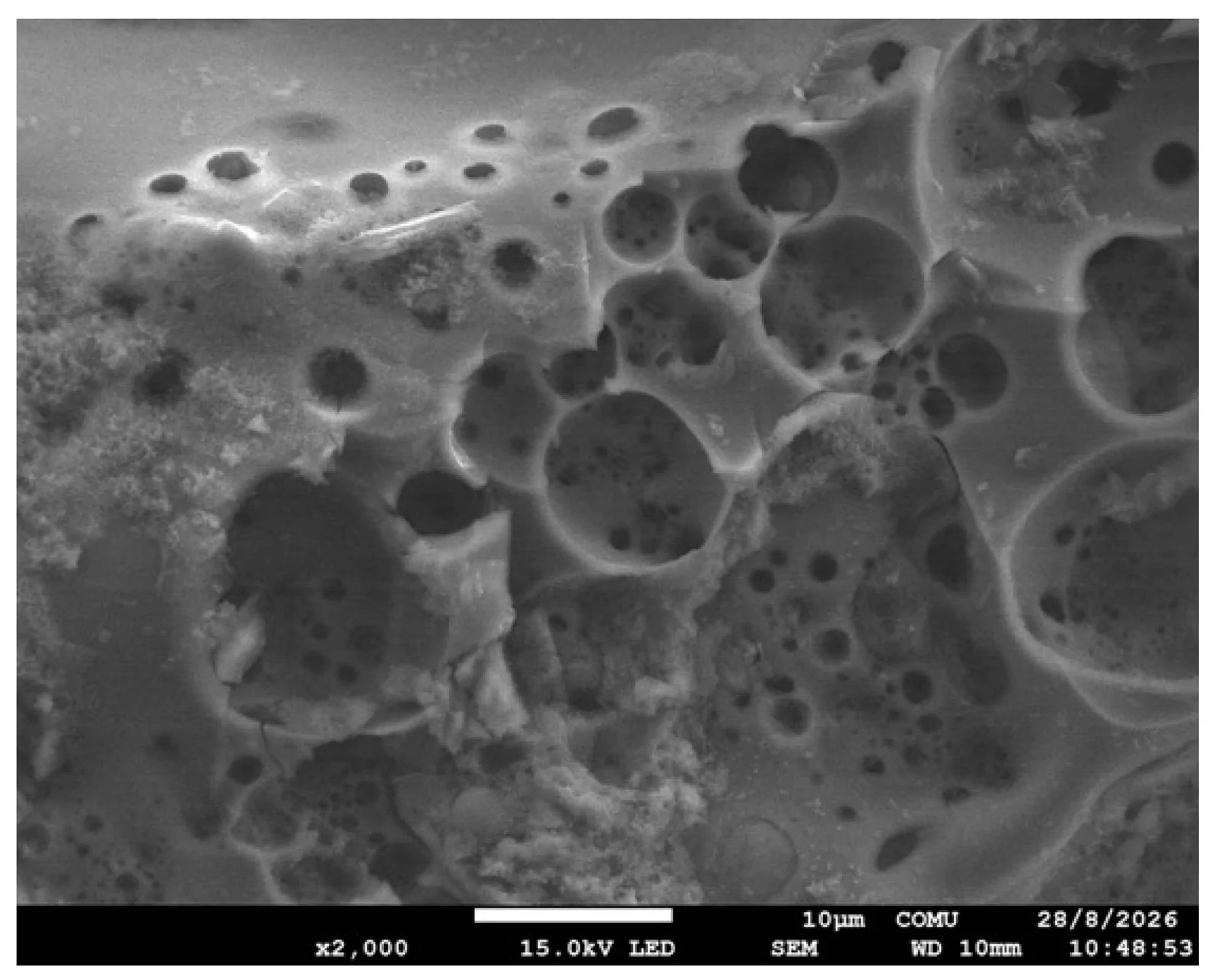
Porous microstructure (x2000).

The water absorption value of the control mixture was determined to be 8.46% at 7 days and 8.47% at 28 days. In contrast, the 7- and 28-day water absorption values of the FA mixture containing fly ash were determined to be 9.40% and 9.75%, respectively. Furthermore, the increase in water absorption values from 7 to 28 days observed in all mixtures containing fly ash is noteworthy. This behavior may be attributed to the fact that the pozzolanic reaction in fly ash begins much later than the hydration of Portland cement [32]. In addition, the 45 µm and 90 µm sieve residue values of the fly ash were 78.4% and 63.6%, respectively, as shown in Table 1, indicating the presence of a considerable amount of coarse particles. The high proportion of coarse particles may exhibit limited reactivity at early ages, resulting in the presence of unreacted or partially reacted particles within the matrix and causing the pore structure to develop differently compared with Portland cement-containing systems. As curing progresses, the continuation of hydration and pozzolanic reactions may lead to changes in pore size distribution and pore connectivity. This may contribute to greater water absorption in the 28-day specimens through capillary pores and interconnected voids [39,40]. In a study conducted by Golewski [1], cement was replaced with Class F fly ash at replacement levels of 0, 20, and 30%, and the corresponding water absorption rates were reported as 5.0, 5.3, and 4.6%, respectively. For the mixture containing 20% fly ash, the hydration products of the concrete were reported to exhibit a loose and porous microstructure, resulting in an increase in the water absorption rate. The aforementioned porous structure is presented in Figure 11.

The addition of CaCl_2_ at a dosage of 1% to the mixtures without fly ash resulted in decreases of 2% and 3% in the 7- and 28-day water absorption values, respectively. However, as the CaCl_2_ dosage increased, the water absorption value subsequently increased, reaching up to 9.8%. In a study conducted by Vilane et al. [38], it was reported that the water absorption rate increased with increasing CaCl_2_ content. The direct relationship between the CaCl_2_ dosage and water absorption capacity can be attributed to the accelerating effect of CaCl_2_ on the hydration process [38]. At a low dosage, CaCl_2_ appears to promote a more compact matrix by significantly enhancing the development of the capillary pore structure at 7 days through accelerated C_3_S and C_3_A hydration processes [30]. The acceleration of cement hydration may increase early-age autogenous shrinkage, resulting in volumetric contraction and consequently the formation of microcracks [41]. The increase in water absorption capacity observed at higher CaCl_2_ dosages may also be associated with the formation of these microcracks.

When 20% of the cement was replaced with fly ash, the 7- and 28-day water absorption values increased by 11% and 15%, respectively. Furthermore, as shown in Figure 10, the incorporation of CaCl_2_ into the fly ash-containing mixtures resulted in a gradual increase in both the 7- and 28-day water absorption values, by approximately 15–43% compared with the control mixture and by 4–24% compared with the mixture containing fly ash alone. The effect of CaCl_2_ on water absorption in fly ash-containing systems can be attributed to the reaction of CaCl_2_ with C_3_A in the hydration environment, resulting in the formation of Friedel’s salt (3CaO•Al_2_O_3_•CaCl_2_•10H_2_O), and consequently a permanent reduction in the amount of available Ca(OH)_2_. The reduction in Ca(OH)_2_ required for the pozzolanic reaction of fly ash further delays the pozzolanic activation mechanism at 28 days, thereby slowing the densification of the pore structure over time [31].

When the effect of TEA addition on the water absorption of the mixtures without fly ash was examined, an approximately 2% decrease in the 7-day water absorption value was observed. For the 28-day water absorption values, decreases of approximately 5.4% were observed when TEA was used at dosages of 0.1% and 0.3%, while a decrease of approximately 2.5% was observed at a TEA dosage of 0.2%. Similarly, the compressive strength results indicated that TEA did not exhibit a response directly proportional to its dosage. This finding suggests that the effect of TEA does not follow a linear trend and that within the scope of the dosages investigated, the determination of an optimum dosage can only be evaluated based on the tested levels. Han et al. [23] reported that the effect of TEA on the pore structure is dosage-dependent and that TEA can reduce pore size at appropriate dosages. When used at optimum dosages, TEA accelerates hydration reactions and promotes the formation of C–S–H gel and AFt/AFm phases, thereby densifying the pore structure of concrete and improving its water absorption behavior [42,43]. However, high TEA dosages may adversely affect the pore structure and increase the volume of large voids, consequently increasing the permeability and water absorption capacity of concrete [42,44].

With the incorporation of fly ash and TEA, the 7- and 28-day water absorption values increased by 12–26% compared with the control mixture. Compared with the FA mixture containing fly ash alone, the addition of TEA at a dosage of 0.1% resulted in a 1.4% increase in the 7-day water absorption value, whereas TEA dosages of 0.2% and 0.3% resulted in decreases of 1% and 2.5%, respectively. However, an increase of 4–10% was observed again in the 28-day water absorption values. This behavior suggests that TEA accelerates hydration at early ages, thereby densifying the pore structure; however, at later ages, TEA may form complexes with Ca^2+^, Al^3+^, and Fe^3+^ ions, and these complex compounds may adversely affect the pozzolanic reactivity of fly ash, potentially resulting in deterioration of the pore structure at later ages [21].

For the mixtures in which CaCl_2_ was used at a dosage of 3% together with TEA at dosages of 0.1, 0.2, and 0.3%, increases of 0.5%, 5.5%, and 3.2%, respectively, were observed in the 7-day water absorption values compared with the control mixture. At 28 days, the use of TEA at dosages of 0.1% and 0.2% resulted in decreases of 2.4% and 0.3%, respectively, whereas the use of TEA at 0.3% resulted in a 2.6% increase in the water absorption value. The increase in water absorption is considered to be primarily attributable to CaCl_2_, whereas the disproportionate variation in the values may be associated with the effect of TEA. Similarly, the 7- and 28-day water absorption values of the mixtures containing fly ash, 3% CaCl_2_, and TEA at dosages of 0.1, 0.2, and 0.3% exhibited a gradual increase of approximately 6–22%. The increase in water absorption observed in the fly ash-containing mixtures is considered to result from the interaction among fly ash, CaCl_2_, and TEA.

When all water absorption findings are evaluated as a whole, the decrease or stabilization of water absorption values over time in the investigated mixtures without fly ash indicates that Portland cement hydration continuously fills the capillary pore network. In all systems containing fly ash, the increase in water absorption values from 7 to 28 days is considered to be associated with the pozzolanic reactivity of fly ash.

### 3.5. ANOVA

The experimental design employed in this study does not constitute a complete CaCl_2_ × TEA × FA factorial design, as not all possible combinations of CaCl_2_ and TEA levels were investigated. The experimental program was designed based on preliminary tests and a subsequent selection of the optimum CaCl_2_ dosage. As the preliminary experiments did not indicate a significant synergistic effect between CaCl_2_ and TEA under the investigated conditions, the full set of factor combinations was not included in the subsequent experimental program. Accordingly, the ANOVA was used to evaluate the main effects of CaCl_2_, TEA, and fly ash (FA) within the investigated experimental design. Interaction terms were not included in the model because the experimental design was not structured to systematically evaluate all possible factor combinations. This approach does not allow the interaction effects to be independently quantified, and the estimated main effects should therefore be interpreted within the limits of the experimental design.

The ANOVA results for the strength values of the mixtures at 1, 3, 7, and 28 days are presented in Table 5. The analysis of variance conducted for curing ages of 1, 3, 7, and 28 days revealed that the relative effects of the factors on strength development varied depending on the curing age. Fly ash was identified as a statistically significant and dominant source of variation at all curing ages (*p* < 0.001). The contribution of fly ash to the total variation was calculated as 85.06%, 90.13%, 86.72% and 78.09% for curing ages of 1, 3, 7, and 28 days, respectively. Particularly at 3 days, the F-value for fly ash reached 198.47, accounting for 90.13% of the total variation. These findings indicate that across the investigated curing ages, the differences in strength were predominantly governed by the fly ash content.

The main effect of CaCl_2_ was not statistically significant compared with fly ash at curing ages of 1, 3, and 7 days (*p* = 0.091, *p* = 0.197 and *p* = 0.165, respectively). However, at the end of the 28-day curing period, the effect of CaCl_2_ became statistically significant (F = 7.14, *p* = 0.005), with its contribution to the total variation increasing to 9.02%. Similarly, the main effect of TEA was not significant compared with fly ash at curing ages of 1, 3, and 7 days (*p* > 0.05), whereas it exhibited a significant effect on the 28-day strength (F = 5.60, *p* = 0.012). The increase in the contribution of TEA to the total variation from 1.69% at 7 days to 7.07% at 28 days indicates that the relative influence of this factor on strength becomes more pronounced at later curing ages.

The ANOVA results indicate that the relative importance of the factors affecting strength varies throughout the curing period. At early ages, fly ash was by far the dominant source of variation, whereas at the end of the 28-day curing period, although the contribution of fly ash decreased relatively, it remained the predominant factor. In contrast, the contributions of CaCl_2_ and TEA increased markedly. Thus, for the 28-day strength, CaCl_2_ and TEA, in addition to fly ash, were identified as statistically significant factors. These findings demonstrate that the effects of the investigated parameters on strength development should not be evaluated solely in terms of constant main effects; rather, their age-dependent effects should also be taken into consideration.

The ANOVA results for the water absorption values of the mixtures at 7 and 28 days are presented in Table 6. The analysis revealed that fly ash was clearly the dominant source of variation among the investigated factors. The effect of fly ash was statistically significant for both the 7-day (F = 161.50, *p* < 0.001) and 28-day (F = 219.91, *p* < 0.001) results, with its contribution to the total variation calculated as 65.08% and 84.57%, respectively. These findings indicate that the variation in water absorption behavior, particularly at the end of the 28-day curing period, was largely attributable to differences in the fly ash content. The increase in the relative contribution of fly ash from 65.08% at 7 days to 84.57% at 28 days demonstrates that with increasing curing age, the proportion of water absorption variability explained by fly ash becomes progressively greater. The CaCl_2_ factor was also found to be statistically significant in the water absorption results at both 7 days (F = 20.10, *p* < 0.001) and 28 days (F = 8.63, *p* = 0.003). However, its contribution to the total variation decreased from 24.30% at 7 days to 9.95% at 28 days. This change indicates that the effect of CaCl_2_ on water absorption was more pronounced at 7 days, whereas the dominance of fly ash increased at later curing ages. In contrast, the effect of TEA was not statistically significant at either 7 days (*p* = 0.228) or 28 days (*p* = 0.557), with its contributions to the total variation being only 2.01% and 0.83%, respectively. Overall, the relative importance of the factors governing water absorption can be ranked as follows: fly ash > CaCl_2_ > TEA. When considered together with the strength results, fly ash appears to be a decisive factor influencing both the mechanical and physical performance of the mixtures. In contrast, CaCl_2_ exerts a statistically significant effect on water absorption at an earlier curing age than on strength, indicating that its influence on water absorption becomes evident earlier during the curing process.

The ANOVA results for the initial and ultimate setting times of the mixtures are presented in Table 7. The ANOVA results for the initial and ultimate setting times revealed that setting behavior exhibited a factor sensitivity markedly different from that observed for strength and water absorption. CaCl_2_ was found to have a statistically significant and dominant effect on the initial setting time (F = 21.93, *p* < 0.001), accounting for 52.55% of the total variation. TEA also exhibited a significant effect on the initial setting time (F = 8.80, *p* = 0.002), with a contribution of 21.09% to the total variation. In contrast, the effect of fly ash was not statistically significant compared with the other parameters (F = 0.09, *p* = 0.769), and its contribution to the total variation remained as low as 0.07%.

A similar factor ranking was observed for the ultimate setting time; however, the relative effect of CaCl_2_ became even more pronounced. With an F-value of 45.08 and *p* < 0.001, CaCl_2_ accounted for 60.18% of the total variation, making it the primary source of variation in setting time. The effect of TEA was also statistically significant (F = 12.58, *p* = 0.001); however, its contribution of 16.80% remained substantially lower than that of CaCl_2_. In contrast, fly ash did not exhibit a statistically significant effect on the ultimate setting time compared with the other parameters (F = 0.02, *p* = 0.880), contributing only 0.01% to the total variation. The increase in the contribution of CaCl_2_ from 52.55% for the initial setting time to 60.18% for the ultimate setting time indicates that CaCl_2_ maintained its dominant influence throughout the later stages of the setting process.

Overall, the relative effects of the factors on setting time can be ranked as follows: CaCl_2_ > TEA > fly ash. This factor sensitivity differs markedly from the fly ash-dominated behavior observed in the strength and water absorption results. Accordingly, while fly ash plays a dominant role in determining the performance properties of the hardened material in the investigated system, setting behavior is primarily governed by the levels of CaCl_2_ and TEA. These findings demonstrate that the effects of the investigated factors should not be evaluated solely from a general “strength-enhancing/strength-reducing” perspective; rather, they should be assessed separately in terms of different performance indicators in both the fresh and hardened states.

The ANOVA results for the initial spread values of the mixtures are presented in Table 8. The effect of fly ash on the initial spread was found to be statistically significant (F = 5.36, *p* = 0.039), accounting for 20.08% of the total variation. In contrast, although CaCl_2_ accounted for the highest proportion of the total variation (29.20%), its effect did not reach statistical significance (F = 2.60, *p* = 0.101). This finding indicates that despite CaCl_2_ explaining a substantial proportion of the variation in the initial spread values, the differences among the factor levels could not be supported by sufficient statistical evidence under the present level of experimental error. The effect of TEA was also not statistically significant (F = 0.87, *p* = 0.486), and its contribution to the total variation remained limited to 9.73%.

Another noteworthy aspect of the initial spread results is that the error variance accounted for 44.98% of the total variation. This proportion is markedly higher than the error contributions observed for strength, water absorption, and setting time, indicating that the initial spread measurements were more susceptible to experimental variability. Therefore, when evaluating the initial spread behavior, not only the contribution ratios of the investigated factors but also the relatively high error variance should be taken into consideration.

The ANOVA results for the relative spread values of the mixtures are presented in Table 9. The analysis showed that none of the investigated factors exhibited a statistically significant main effect at the 5% significance level. Although CaCl_2_ had the highest F-value among the investigated factors (F = 3.08, *p* = 0.068), its effect did not reach statistical significance. Nevertheless, the contribution of CaCl_2_ to the total variation was 37.99%, which was markedly higher than those of the other factors. This finding indicates that although CaCl_2_ accounted for a substantial proportion of the variation in relative spread, the differences among the CaCl_2_ levels could not be supported by sufficient statistical evidence under the present level of experimental error.

The effect of TEA was found to be rather limited, with an F-value of 0.10 and *p* = 0.961, accounting for only 1.18% of the total variation. Similarly, the effect of fly ash was not statistically significant (F = 1.02, *p* = 0.334), with a contribution of 4.17% to the total variation. Notably, although the effect of fly ash was statistically significant for the initial spread (*p* = 0.039), it did not retain statistical significance for the relative spread (*p* = 0.334). This finding indicates that the effect of fly ash on absolute spread behavior was not reflected to the same extent in the relative spread indicator.

The fact that the error variance accounted for 49.28% of the total variation in the relative spread results indicates that this parameter was more sensitive to experimental variability than to the investigated factors. Therefore, when evaluating the effects of the factors on relative spread, the high contribution of error variance should be considered alongside statistical significance. Overall, although CaCl_2_ had the highest contribution to the variation in relative spread, none of the investigated factors—CaCl_2_, TEA or fly ash—exhibited a statistically significant main effect.

It should be noted that the PCE dosage was not kept constant across all mixtures. A dosage of 1.0 g was used in the mortars without fly ash, whereas 2.5 g was used in all mortars containing 20% fly ash. This adjustment was made to account for the differences in mixture behavior associated with the incorporation of fly ash. However, because the PCE dosage changed simultaneously with the fly ash content, the individual contributions of these two variables cannot be fully separated within the present experimental design. Consequently, although the ANOVA results identify fly ash as a statistically significant factor accounting for a large proportion of the observed variation in several properties, the estimated contribution of fly ash should be interpreted with this limitation in mind. In particular, part of the observed differences between mixtures with and without fly ash may also be associated with the change in PCE dosage. Therefore, the reported contribution percentages represent the effects associated with the investigated factor configuration and should not be interpreted as definitive evidence of the isolated effect of fly ash.

## 4. Conclusions

Within the scope of this study, the effects of fly ash, CaCl_2_, and TEA on the fresh and hardened properties of cementitious systems were comprehensively investigated. By evaluating the results obtained for setting time, spread, compressive strength, and water absorption together with the ANOVA analyses, the effects of admixture type and dosage on mixture performance were elucidated. The main findings obtained from the study are presented below:

(1) While the use of fly ash delayed the setting time, the addition of CaCl_2_ significantly shortened the setting time with increasing dosage and reduced the retarding effect of fly ash. In contrast, TEA prolonged the setting time, particularly at higher dosages; however, when used in combination with CaCl_2_, this retarding effect was largely counterbalanced.
(2) It was determined that the loss of consistency varied depending on the type and dosage of the admixture. While CaCl_2_ rapidly reduced the spread values, particularly at high dosages, TEA exhibited dosage-dependent variable behavior when used alone and significantly slowed the loss of consistency when used in combination with CaCl_2_. The use of fly ash, particularly in the CaCl_2_–TEA combination, helped maintain workability for a longer period.
(3) The compressive strength results demonstrated that the effects of the admixtures were strongly dependent on both their dosage and the binder composition. In mixtures without fly ash, 1% CaCl_2_ produced the most favorable results in terms of both early- and later-age strength, whereas higher dosages of CaCl_2_ and TEA adversely affected the strength. In fly ash-containing mixtures, the individual or combined use of CaCl_2_ and TEA generally resulted in a reduction in strength, with the CaCl_2_–TEA combination yielding the lowest strength values. These findings indicate that in fly ash-containing systems, the dosage of chemical admixtures and their interactions with one another are critical factors governing strength development.
(4) The water absorption results showed that the use of fly ash increased the water absorption rate by creating a more porous structure, and this effect became more pronounced at 28 days. In systems without fly ash, low dosages of CaCl_2_ and TEA limited water absorption, whereas high CaCl_2_ dosages and CaCl_2_–TEA combinations containing fly ash increased the water absorption values. Overall, it was determined that the effects of chemical admixtures on pore structure in fly ash-containing systems varied depending on the dosage and the interaction between the admixtures.

## Figures and Tables

**Table 1:** Chemical composition of cement and fly ash.

Oxide (%)	Cement	Fly Ash
SiO_2_	19.02	51.14
Al_2_O_3_	4.43	25.45
Fe_2_O_3_	2.93	9.32
CaO	62.46	6.46
MgO	2.81	1.27
SO_3_	3.40	0.61
Na_2_O	0.77	0.73
K_2_O	0.77	1.01
Cl	0.05	0.04
Others	0.41	4.01
Loss on ignition	3.43	0.82
45 on the micron sieve	4.2	78.4
90 on the micron sieve	0.4	63.6
Specific surface (cm^2^/g)	4610	5170
Specific gravity (g/cm^3^)	3.20	2.43

**Table 2:** Some physical and chemical properties of chemical admixtures.

Admixture	Alkali Content (%)	Intensity (g/cm^3^)	Solid Content (%)	Chloride Content (%)	pH, 25 °C	Physical State
CaCl_2_	-	2.150	-	64	-	White, granular, odorless
TEA	-	1.125	-	-	10.5	Pale yellow viscous liquid
PCE	4.5	1.047	21.44	0.05	3.65	Brown, liquid

**Table 3:** Mix proportions for the paste mixtures.

Sample	Cement (g)	Fly Ash (g)	Water (g)	CaCl_2_ (g)	TEA (g)
C	500	0	200	0	0
CC1	500	0	200	5	0
CC3	500	0	200	15	0
CC5	500	0	200	25	0
TEA01	500	0	200	0	0.5
TEA02	500	0	200	0	1
TEA03	500	0	200	0	1.5
CC3-TEA01	500	0	200	15	0.5
CC3-TEA02	500	0	200	15	1
CC3-TEA03	500	0	200	15	1.5
FA	400	100	200	0	0
FA-CC1	400	100	200	5	0
FA-CC3	400	100	200	15	0
FA-CC5	400	100	200	25	0
FA-TEA01	400	100	200	0	0.5
FA-TEA02	400	100	200	0	1
FA-TEA03	400	100	200	0	1.5
FA-CC3-TEA01	400	100	200	15	0.5
FA-CC3-TEA02	400	100	200	15	1
FA-CC3-TEA03	400	100	200	15	1.5

**Table 4:** Mix proportions for the mortar mixtures.

Sample	Cement (g)	Fly Ash (g)	Water (g)	Sand (g)	CaCl_2_ (g)	TEA (g)	PCE (g)	Flow (cm)
C	500	0	242.5	1375	0	0	1	18.5
CC1	500	0	242.5	1375	5	0	1	19.5
CC3	500	0	242.5	1375	15	0	1	19.0
CC5	500	0	242.5	1375	25	0	1	18.5
TEA01	500	0	242.5	1375	0	0.5	1	19.5
TEA02	500	0	242.5	1375	0	1	1	19.0
TEA03	500	0	242.5	1375	0	1.5	1	19.0
CC3-TEA01	500	0	242.5	1375	15	0.5	1	19.0
CC3-TEA02	500	0	242.5	1375	15	1	1	19.0
CC3-TEA03	500	0	242.5	1375	15	1.5	1	19.5
FA	400	100	242.5	1375	0	0	2.5	17.5
FA-CC1	400	100	242.5	1375	5	0	2.5	18.0
FA-CC3	400	100	242.5	1375	15	0	2.5	18.0
FA-CC5	400	100	242.5	1375	25	0	2.5	19.0
FA-TEA01	400	100	242.5	1375	0	0.5	2.5	17.0
FA-TEA02	400	100	242.5	1375	0	1	2.5	18.0
FA-TEA03	400	100	242.5	1375	0	1.5	2.5	17.5
FA-CC3-TEA01	400	100	242.5	1375	15	0.5	2.5	19.5
FA-CC3-TEA02	400	100	242.5	1375	15	1	2.5	19.0
FA-CC3-TEA03	400	100	242.5	1375	15	1.5	2.5	20.0

**Table 5:** ANOVA results of the 1-, 3-, 7-, and 28-day strength values of the mixtures.

Source	Sum of Squares (SS)	Degrees of Freedom (d_f_)	Variance (V)	F	*p* Value	Contribution (%)
**1-day**						
CC	41	3	13.58	2.73	0.091	5.44%
TEA	15	3	5	1.00	0.425	2.00%
FA	637	1	636.54	127.78	0.000	85.06%
Error	60	12	4.98			7.99%
Total	748	19				
**3-day**						
CC	32	3	10.50	1.82	0.197	2.48%
TEA	36	3	11.96	2.08	0.157	2.83%
FA	1143	1	1142.79	198.47	0.000	90.13%
Error	69	12	5.76			5.45%
Total	1268	19				
**7-day**						
CC	70	3	23.44	2.02	0.165	3.71%
TEA	32	3	10.66	0.92	0.461	1.69%
FA	1644	1	1643.90	141.59	0.000	86.72%
Error	139	12	11.61			7.35%
Total	1896	19				
**28-day**						
CC	187	3	62.44	7.14	0.005	9.02%
TEA	147	3	48.98	5.60	0.012	7.07%
FA	1622	1	1622.18	185.56	0.000	78.09%
Error	105	12	8.74			5.05%
Total	2077	19				

**Table 6:** ANOVA results of the 7-day and 28-day water absorption values of the mixtures.

Source	Sum of Squares (SS)	Degrees of Freedom (df)	Variance (V)	F	*p* Value	Contribution (%)
**7-day**						
CC	3	3	1.05	20.10	0.000	24.30%
TEA	0	3	0.09	1.66	0.228	2.01%
FA	9	1	8.46	161.50	0.000	65.08%
Error	1	12	0.05			4.84%
Total	13	19				
**28-day**						
CC	4	3	1.38	8.63	0.003	9.95%
TEA	0	3	0.12	0.72	0.557	0.83%
FA	35	1	35.25	219.91	0.000	84.57%
Error	2	12	0.16			4.62%
Total	42	19				

**Table 7:** ANOVA results for the initial and ultimate setting times of the mixtures.

Source	Sum of Squares (SS)	Degrees of Freedom (df)	Variance (V)	F	*p* Value	Contribution (%)
**Initial Setting Time**						
CC	44,516	3	14,839	21.93	0.000	52.55%
TEA	17,869	3	5956	8.80	0.002	21.09%
FA	61	1	61	0.09	0.769	0.07%
Error	8120	12	677			9.59%
Total	84,714	19				
**Ultimate Setting Time**						
CC	55,651	3	18,550	45.08	0.000	60.18%
TEA	15,530	3	5177	12.58	0.001	16.80%
FA	10	1	10	0.02	0.880	0.01%
Error	4938	12	412			5.34%
Total	92,476	19				

**Table 8:** ANOVA results of the initial spread values of the mixtures.

Source	Sum of Squares (SS)	Degrees of Freedom (df)	Variance (V)	F	*p* Value	Contribution (%)
CC	4	3	1.19	2.60	0.101	29.20%
TEA	1	3	0.40	0.87	0.486	9.73%
FA	3	1	2.45	5.36	0.039	20.08%
Error	6	12	0.46			44.98%
Total	12	19				

**Table 9:** ANOVA results regarding the relative spread values of the mixtures.

Source	Sum of Squares (SS)	Degrees of Freedom (df)	Variance (V)	F	*p* Value	Contribution (%)
CC	0.069	3	0.023	3.08	0.068	37.99%
TEA	0.002	3	0.001	0.10	0.961	1.18%
FA	0.008	1	0.008	1.02	0.334	4.17%
Error	0.089	12	0.007			49.29%
Total	0.181	19				

## Data Availability

The original contributions presented in this study are included in the article. Further inquiries can be directed to the corresponding author.
